# Association of protein kinase CK2 inhibition with cellular radiosensitivity of non-small cell lung cancer

**DOI:** 10.1038/s41598-017-16012-1

**Published:** 2017-11-23

**Authors:** Qianwen Li, Ke Li, Tianyang Yang, Sheng Zhang, Yu Zhou, Zhenyu Li, Jinrong Xiong, Fangzheng Zhou, Xiaoshu Zhou, Li Liu, Rui Meng, Gang Wu

**Affiliations:** 1 Cancer Center, Union Hospital, Tongji Medical College, Huazhong University of Science and Technology, Wuhan, 430022 China; 2 Department of Pharmacy, Union Hospital, Tongji Medical College, Huazhong University of Science and Technology, Wuhan, 430022 China; 3 Department of Nuclear Medicine, Tongji Hospital, Tongji Medical College, Huazhong University of Science and Technology, Wuhan, 430030 China; 4 Oncology Department, The Chinese People’s Liberation Army 457 Hospital, Wuhan, 430012 China

## Abstract

Protein kinase CK2 is a highly conserved protein Ser/Thr protein kinase and plays important roles in cell proliferation, protein translation and cell survival. This study investigated the possibility of using CK2 inhibition as a new approach for increasing the efficacy of radiotherapy in non-small cell lung cancer (NSCLC) and its underlying mechanisms. Kinase inhibition of CK2 was attempted either by using the specific CK2 inhibitor, Quinalizarin or by applying siRNA interference technology to silence the expression of the catalytic subunit of CK2 in A549 and H460 cells. The results showed that CK2α knockdown or Quinalizarin significantly enhanced the radiosensitivity of various NSCLC cells. The notable findings we observed after exposure to both CK2 inhibition and ionizing radiation (IR) were a prolonged delay in radiation-induced DNA double-strand breaks (DSB) repair, robust G2/M checkpoint arrest and increased apoptosis. *In vivo* studies further demonstrated that compared with each treatment alone, CK2 inhibition combined with IR reduced tumor growth in the H460 cell xenograft model. In conclusion, CK2 is a promising target for the enhancement of radiosensitivity in NSCLC.

## Introduction

Lung cancer is the leading cause of cancer deaths worldwide, affecting approximately 1.6 million people worldwide each year^1^. Radiation therapy is commonly used in lung cancer treatment for either curative or palliative purposes. However, the intrinsic radioresistance of cancer cells limits its efficacy, leading to tumor recurrences in the previously irradiated field^2^. Hence, elucidating the mechanism of radioresistance in lung cancer cells is a key topic demanding prompt solution.

Protein kinase CK2 is a highly conserved protein Ser/Thr protein kinase, consisting of 2 catalytic (α α, α′α′ or α α′) subunits and 2 regulatory (β β) subunits^3–5^. Studies have revealed that overexpression of CK2 has been associated with the promotion of tumorigenesis in the lung^6^, and the activity of CK2 was up to 2–3 folds higher in lung cancer cells than in normal lung tissues^7^.

There are more than 300 CK2 substrates, which played vital roles in cell proliferation, apoptosis and DNA damage repair regulation processes^8–11^. It is noteworthy that some of these CK2 substrates are key molecules involved in the major cellular processes after radiation. These include XRCC4 and MDC1, which played key roles in the DNA double-stand breaks repair process and are phosphorylated and regulated by CK2^12,13^. Moreover, P53, a vital molecule in cell apoptosis and cell cycle arrests, are an important substrate of CK2^14^.

Therefore, it is logical to propose that CK2 is an essential target in regulating the important cell processes after radiation^5,15^. In this study, we investigated the radiosensitizing effect of the down regulation of CK2 in various lung cancer cell lines and tried to identify the underlying mechanisms using *in vivo* and *in vitro* experiments.

## Results

### CK2 is ubiquitously expressed in lung cancer cells and tissues

**Figure 1:**
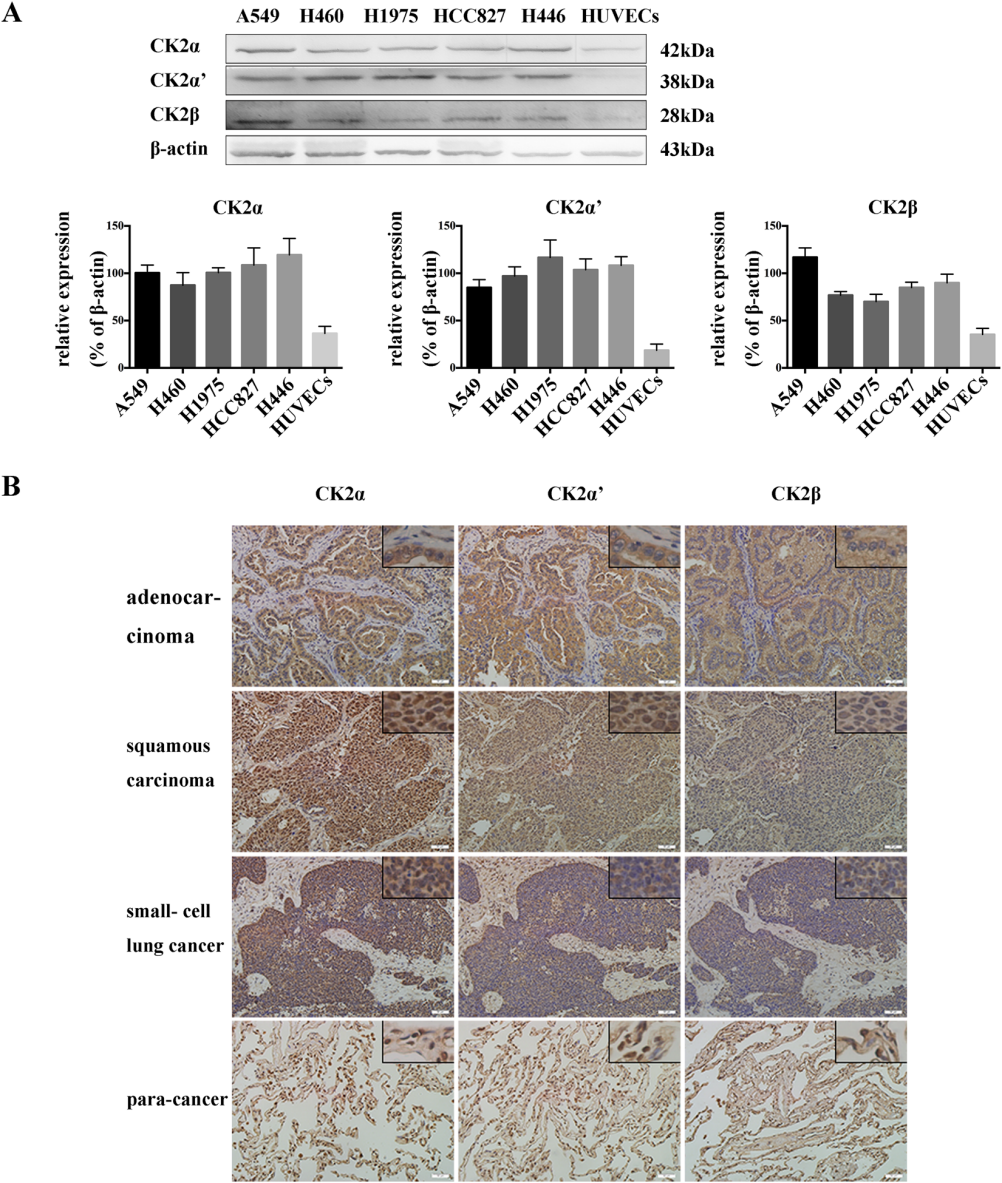
CK2 expression in human lung cancer cells and tissues. (**A**) The protein expression of CK2α,α′ and β in different types of lung cancer cells were examined by Western blot. (**B**) Expression of CK2α, α′ and β in different types of lung cancer and para-cancerous tissues were measured by immunohistochemistry. Images were taken at 200 × and 400 × magnification.

To evaluate the role of CK2 in regulating the radiosensitivity of lung cancers after ionizing radiation (IR), we first examined the protein expression of CK2 subunits in different human lung cancer cells and HUVECs cells by Western blot. A549 cells are EGFR wild type adenocarcinoma cells, while H1975 (EGFR L858R + T790M) and HCC827 (EGFR E716-A750del) cells are EGFR mutant adenocarcinoma cells. H460 cells are large cell lung cancer cells and H446 cells are small cell lung cancer cells, and HUVECs are normal endothelial cells. As shown in Fig. 1A, the three subunits of CK2 were expressed in all these types of lung cancer cells, and the protein amount was relatively higher than that in non-cancerous HUVECs.

In addition to detecting the CK2 protein expression in the established cell lines, we also stained the lung cancer tissues of various pathological subtypes as well as their adjacent para-neoplastic normal lung tissues with specific CK2α, α′ and β antibodies (Fig. 1B). The results showed that all of the subunits were gathered in cytoplasm in the adenocarcinoma. CK2α was distributed in both the cytoplasm and nucleus, while CK2α′ presented mainly in the cytoplasm, except for a small amount in the nucleus. CK2β was expressed in the cytoplasm in squamous carcinoma and small- cell lung cancer tissues. In the para-neoplastic tissues, CK2α, α′ and β all expressed in the cytoplasm and part of the nucleus. Above all, CK2 α, α′ and β were ubiquitously presented in lung cancer tissues with a relatively elevated protein level compared with that in adjacent para-neoplastic tissues.

### Quinalizarin, a specific CK2 inhibitor, reduces CK2 kinase activity in lung cancer cells

**Figure 2:**
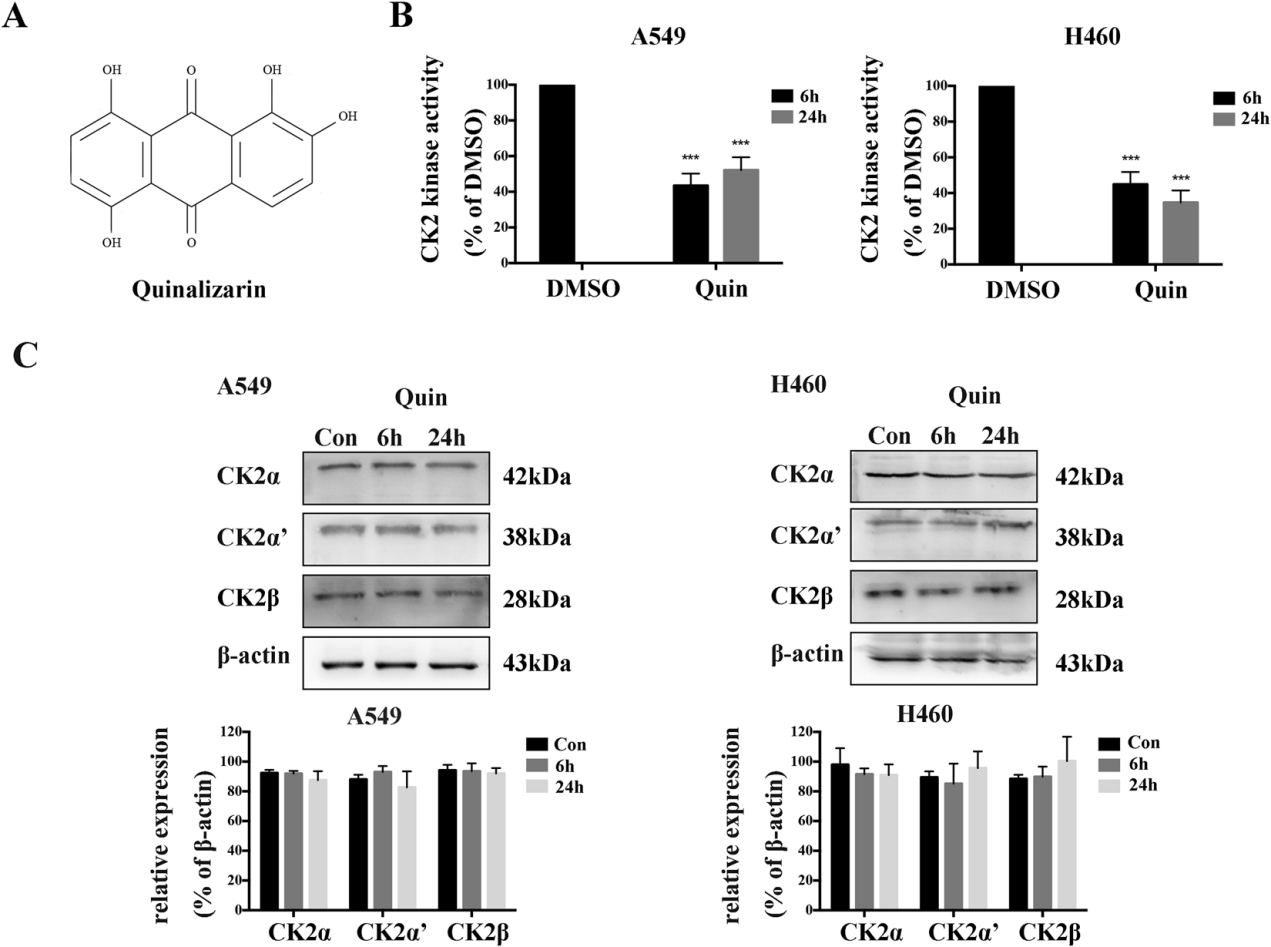
Quinalizarin reduces the CK2 kinase activity in lung cancer cells. (**A**) The chemical structure of Quinalizarin. (**B**) A549 or H460 cells were treated with DMSO or 25 μM Quinalizarin for 6 or 24 h. The CK2 kinase activity after inhibition was measured by the kinase assay. Proteins were extracted and the CK2 activity was determined using the incorporation of 32P-phosphate into the synthetic substrate RRRDDDSDDD. The cells treated with the solvent DMSO alone were used as a control and the activity was set to 100%. (***p < 0.001) The mean ± S.D. was calculated for three independent experiments. (**C**) The protein expression of CK2α,α’ and β was measured by Western blot. The mean ± S.D. was calculated for three independent experiments.

To analyze the role of CK2 in lung cancer cells after IR, we suppressed the CK2 kinase activity with, to date, one of the most specific CK2 inhibitors to date, Quinalizarin. Over the last 20 years, numerous inhibitors for the protein kinase CK2 has been reported in the literature. It turned out that Quinalizarin (Fig. 2A) is a very effective and rather specific inhibitor of CK2^16,17^. To test the inhibitory effect of Quinalizarin on the CK2 kinase activity in lung cancer cells, we conducted the kinase assay on A549 and H460 cells. The cells were treated with 25 μM Quinalizarin for either 6 or 24 h, the CK2 kinase assay was then performed. As shown in Fig. 2B,C, it could clearly be seen that Quinalizarin inhibited CK2 kinase activity at a concentration of 25 μM, and the CK2 kinase activity was reduced by approximately 40% to 60% after 6 and 24 h of treatment, without affecting the protein amounts of all three CK2 subunits. All subsequent experiments with this inhibitor were mostly performed mostly at 25 μM concentration for up to 24 h.

### Down regulation of CK2 suppresses the cell viability and enhances the radiosensitivity of lung cancer cells

**Figure 3:**
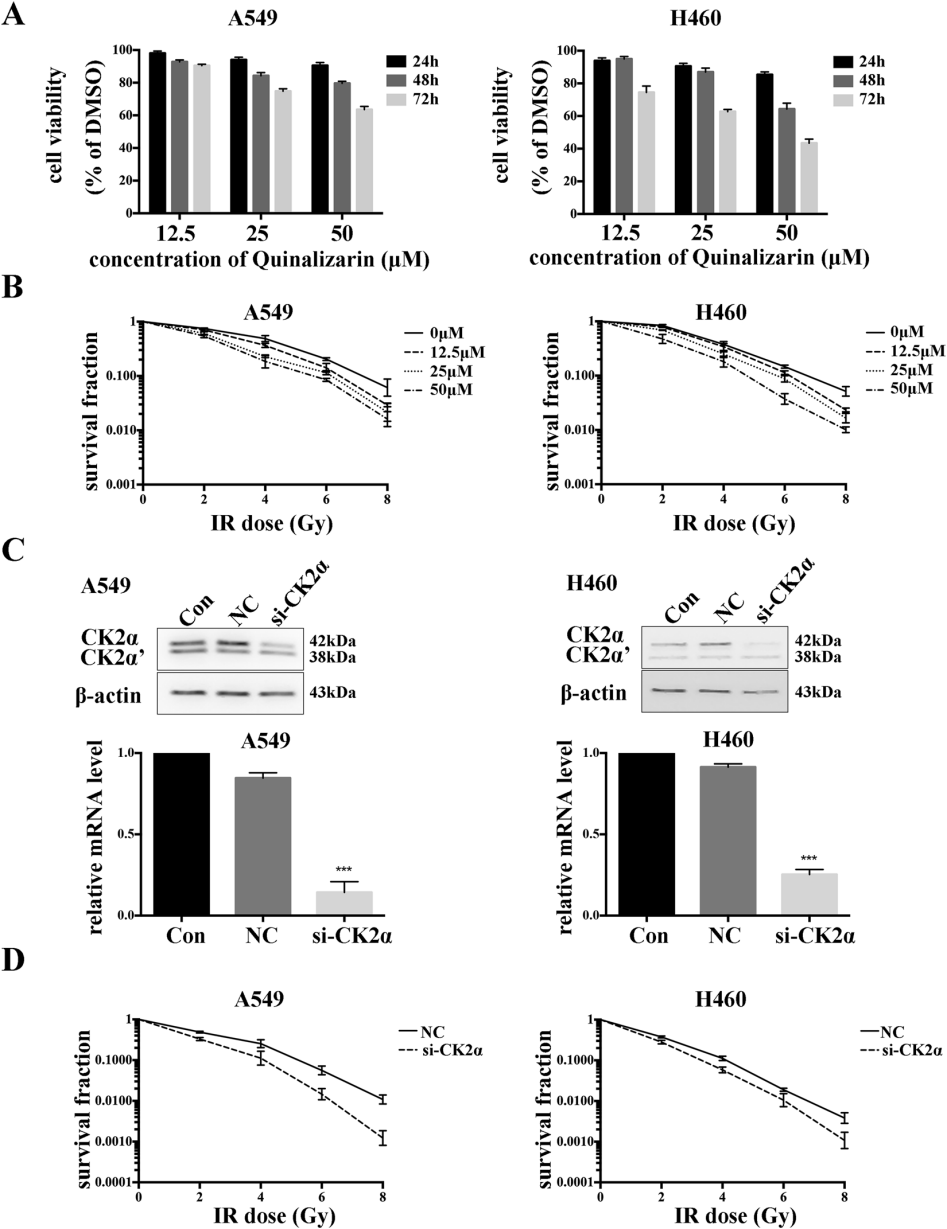
Down regulation of CK2 suppressed the cell viability and enhance the radiosensitivity of lung cancer cells. (**A**) A549 or H460 cells were treated with DMSO or 12.5, 25, or 50 μM Quinalizarin for 24, 48 or 72 h. Cell viability was measured using the CCK8 assay. DMSO-treated cells were taken as a control. The mean ± S.D. was calculated for three independent experiments. (**B**) Cells were treated with DMSO or indicated concentrations of Quinalizarin for 24 h, then exposed to 0, 2, 4, 6, or 8 Gy of X-ray radiation. The culture medium was changed to the fresh one 24 h after IR. After further incubation for 14 days, the cells were fixed and stained with Giemsa, and colonies containing no less than 50 cells were counted as a clone. The mean ± S.D. was calculated for three independent experiments. (**C**) Cells were transfected with a negative control siRNA or si-CK2α. Total protein and mRNA were extracted after 48 h, and the protein expression and mRNA level of CK2α were detected by Western bolt and RT-PCR respectively. (***p < 0.001) The mean ± S.D. was calculated for three independent experiments. (**D**) IR was conducted 48 h after transfection at different doses (0, 2, 4, 6, or 8 Gy) and radiosensitivity was measured using a colony formation assay. The mean ± S.D. was calculated for three independent experiments.

For determination of the cell viability, different concentrations of Quinalizarin were used to treat A549 and H460 cells for 24, 48 or 72 h. The results of the CCK8 assays showed that Quinalizarin could suppress the viability of the cells in a concentration and time dependent manner (Fig. 3A). At the 24 h time point, the inhibitory rates were less than 25% under all three concentrations, while at the 25 μM concentration, the viability for A549 and H460 cells were 93.99 ± 1.53% (p = 0.0025) and 90.52 ± 1.78% (p = 0.0008), respectively, in contrast to the DMSO group. At the 48 and 72 h time point or at the 50 μM concentration, the cell viability decreased sharply, with the 50 μM and 72 h viability reaching 63.54 ± 1.94% (p < 0.0001) in A549 and 43.38 ± 2.50% (p < 0.0001) in H460 cells respectively.

To explore the impact of Quinalizarin on the radiation response, A549 and H460 cells were treated with DMSO or 12.5, 25, or 50 μM Quinalizarin, and then exposed to 0, 2, 4, 6, or 8 Gy X-ray radiation. After further incubation for 10–14 days, the clones were counted. As shown in Fig. 3B, a lower survival fraction in both the cell lines was seen when treated with a higher concentration of Quinalizarin after exposure to different IR doses. Compared with the DMSO control group, when treated with 25 μM Quinalizarin, the values of SF_2_ (cell survival fraction at 2 Gy) for the A549 and H460 cells were decreased from 0.73 ± 0.02 to 0.64 ± 0.04 (p = 0.0252) and from 0.84 ± 0.04 to 0.69 ± 0.01 (p = 0.0032), respectively, suggesting radiosensitivity enhancement.

Since the EGFR mutation is a clinical significant molecular characteristic of lung cancer, the effect of Quinalizarin on the radiosensitivity of the EGFR mutant adenocarcinoma cells H1975 and HCC87 was also explored in the current study. As shown in supplementary Fig. S1, a similar decreased SF_2_ value was observed. Furthermore, in small cell lung cancer H446 cells, the SF_2_ value was also reduced (supplementary Fig. S1) after treatment with Quinalizarin.

To further prove that the radiosensitizing effect of Quinalizarin on lung cancer cells was specifically due to the inhibition of CK2, A549 and H460 cells were transfected with si-CK2α or negative control siRNA, and the protein and mRNA levels of CK2α were measured 48 h later to ensure the transfection efficiency (Fig. 3C). Colony formation assays were conducted to determine the radiosensitizing effect of CK2α depletion on A549 and H460 (Fig. 3D). The reduction of SF_2_ from 0.49 ± 0.01 to 0.34 ± 0.02 (p = 0.0003) in A549 cells and from 0.37 ± 0.01 to 0.29 ± 0.02 (p = 0.0034) in H460 cells indicated that specifical down regulation of CK2α by siRNA could significantly enhance the radiosensitivity of A549 and H460 cells. Thus, we concluded that kinase inhibition of CK2 either by using the specific inhibitor Quinalizarin or by applying si-CK2α to silence the expression of the catalytic subunit of CK2, could significantly enhance the radiosensitivity of lung cancer cells in the A549 and H460.

### Effect of down regulation of CK2 on IR induced apoptosis and cell cycle distribution

**Figure 4:**
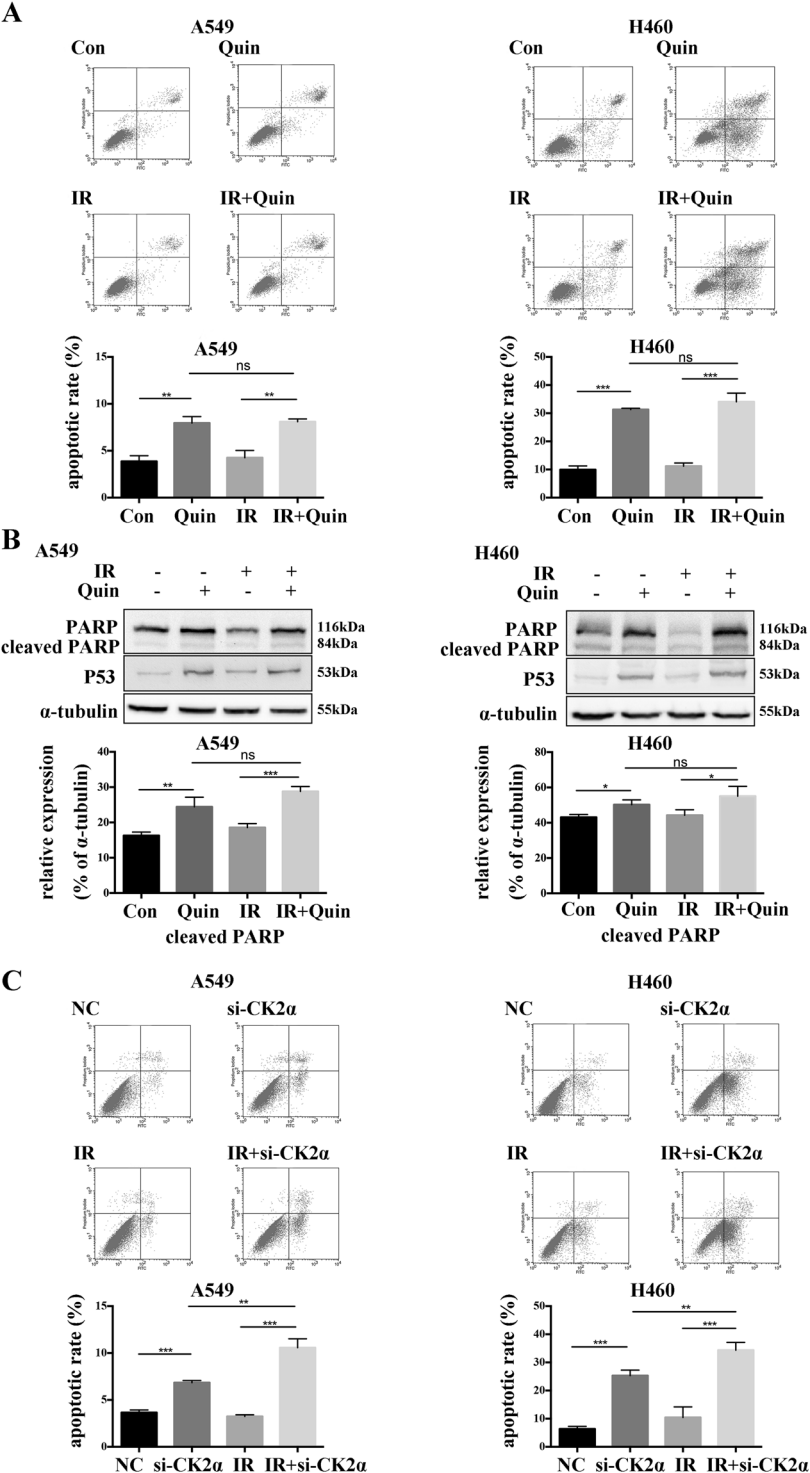
Effect of the down regulation of CK2 on IR-induced apoptosis. (**A**) A549 or H460 cells were pretreated with DMSO or 25 μM Quinalizarin for 24 h, then exposed to 0 or 4 Gy X-ray radiation. After 24 h, the cells were collected, resuspended with binding buffer and then stained with Annexin-V-PE and 7-AAD. Finally, apoptosis was measured by flow cytometry. The apoptosis rates were calculated. (**p < 0.01, ***p < 0.001) The mean ± S.D. was calculated for three independent experiments. (**B**) Cells were pretreated with DMSO or 25 μM Quinalizarin for 24 h, then exposed to 0 or 4 Gy X-ray radiation. After 24 h, the cells were harvested and total proteins were extracted. The protein level of PARP and P53 was detected by Western blot. (**C**) Cells were transfected with negative control siRNA or si-CK2α, after 48 h cells were exposed to 0 or 4 Gy X-ray radiation. 24 h later, the cells were collected and apoptosis was measured by flow cytometry. The apoptosis rates were calculated. (**p < 0.01, ***p < 0.001) The mean ± S.D. was calculated for three independent experiments.

To examine the effect of the down regulation of CK2 on cell apoptosis in A549 and H460 cells after exposure to IR, flow cytometry assays were employed. As shown in Fig. 4A, the down regulation of CK2 by Quinalizarin increased the apoptosis rates from 3.87 ± 0.60% to 7.95 ± 0.70% in A549 cells and 9.92 ± 1.36% to 31.32 ± 0.46% in H460 cells. The combination of IR and Quinalizarin (8.1 ± 0.29% in A549 and 34.06 ± 3.08% in H460) had a higher apoptosis rate than IR alone (4.25 ± 0.78% in A549 and 11.20 ± 1.12% in H460), However, it failed to enhance the apoptotic rate in contrast to Quinalizarin alone (7.95 ± 0.70% in A549 and 31.32 ± 0.46% in H460). Western blot results showed that Quinalizarin increased the expression of cleaved PARP and P53 with or without IR (Fig. 4B). On the other hand, cells transfected with si-CK2α showed a significantly increased apoptotic rate from 3.65 ± 0.28% to 6.85 ± 0.22% in A549 cells and 6.34 ± 0.95% to 25.3 ± 1.93% in H460 cells. In addition, the combination of IR and si-CK2α (10.57 ± 0.95% in A549 and 34.34 ± 2.75% in H460) had a higher apoptotic rate than si-CK2α or IR (3.23 ± 0.18% in A549 and 10.45 ± 3.72% in H460) alone (Fig. 4C). Collectively, the increase in apoptosis enhanced radiosensitivity through si-CK2α but not Quinalizarin.

**Figure 5:**
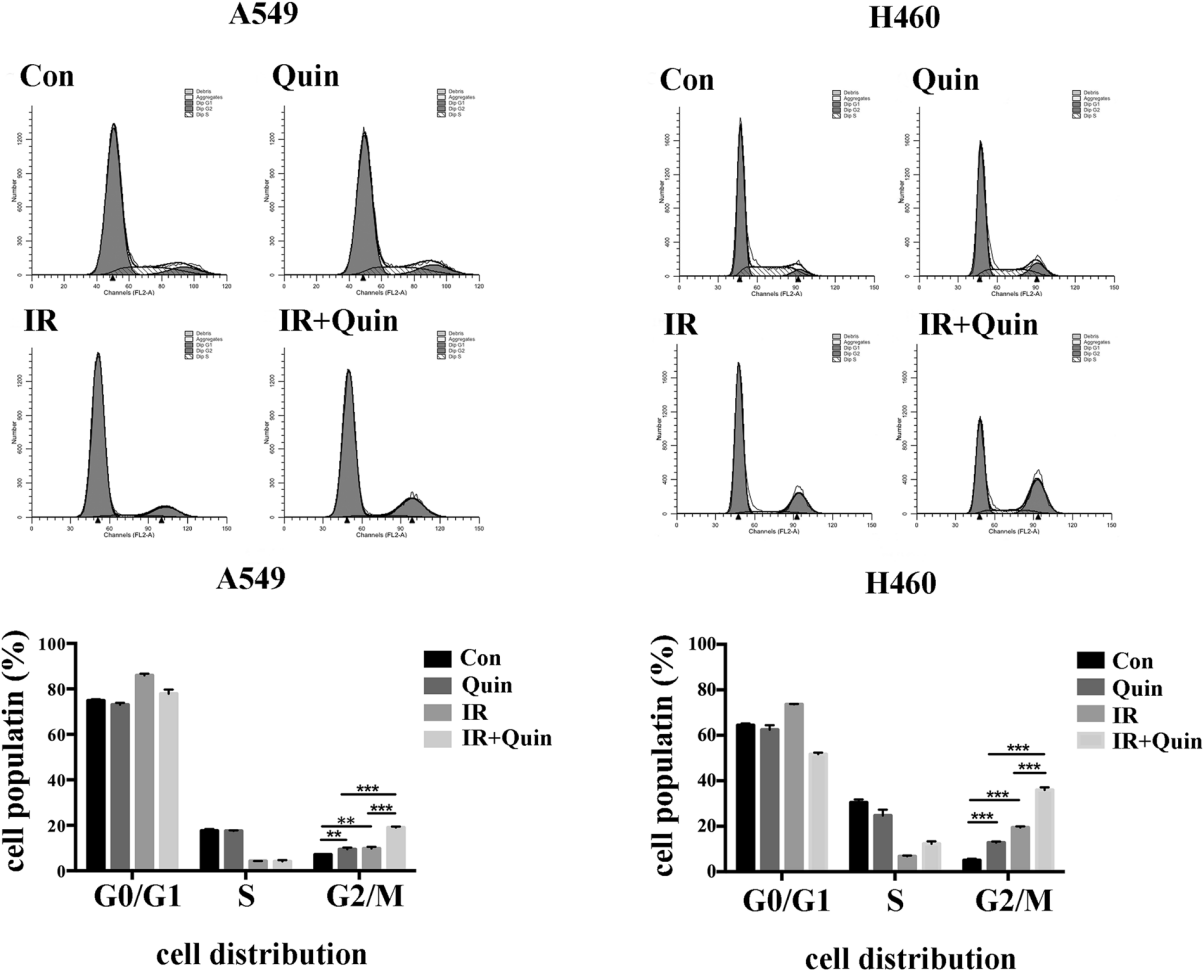
The effect of down regulation of CK2 on the IR-induced cell cycle distribution. A549 or H460 cells were pretreated with DMSO or 25 μM Quinalizarin for 24 h, then exposed to 0 or 4 Gy X-ray radiation. The cells were collected 24 h after IR exposure, fixed overnight, stained with RNase and propidium iodide and then the cell cycle distribution was measured by flow cytometry. Cell populations were calculated. (**p < 0.01 and ***p < 0.001) The mean ± S.D. was calculated for three independent experiments.

To further investigate the potential mechanism of radiosensition by Quinalizarin, we measured the cell cycle distribution. It is well known that the cell cycle distribution influences the cell radiosensitivity and cells in the G2/M phase are the most sensitive to IR. In the present study, cells that were treated with Quinalizarin alone, compared with the DMSO control, showed increasing G2/M accumulation in both A549 (7.16 ± 0.08% vs 9.46 ± 0.76%) and H460 cells (5.04 ± 0.64% vs 12.82 ± 0.48%). In the combination group, we observed a significant increase in the cell population in G2/M phase (19.01 ± 0.46% in A549 and 35.94 ± 1.21% in H460) compared with that in Quinalizarin group (9.46 ± 0.76% in A549 and 12.82 ± 0.48% in H460) or IR group (9.79 ± 0.76% in A549 and 19.50 ± 0.44% in H460) (Fig. 5).

### Effect of down regulation of CK2 and IR on cell proliferation

**Figure 6:**
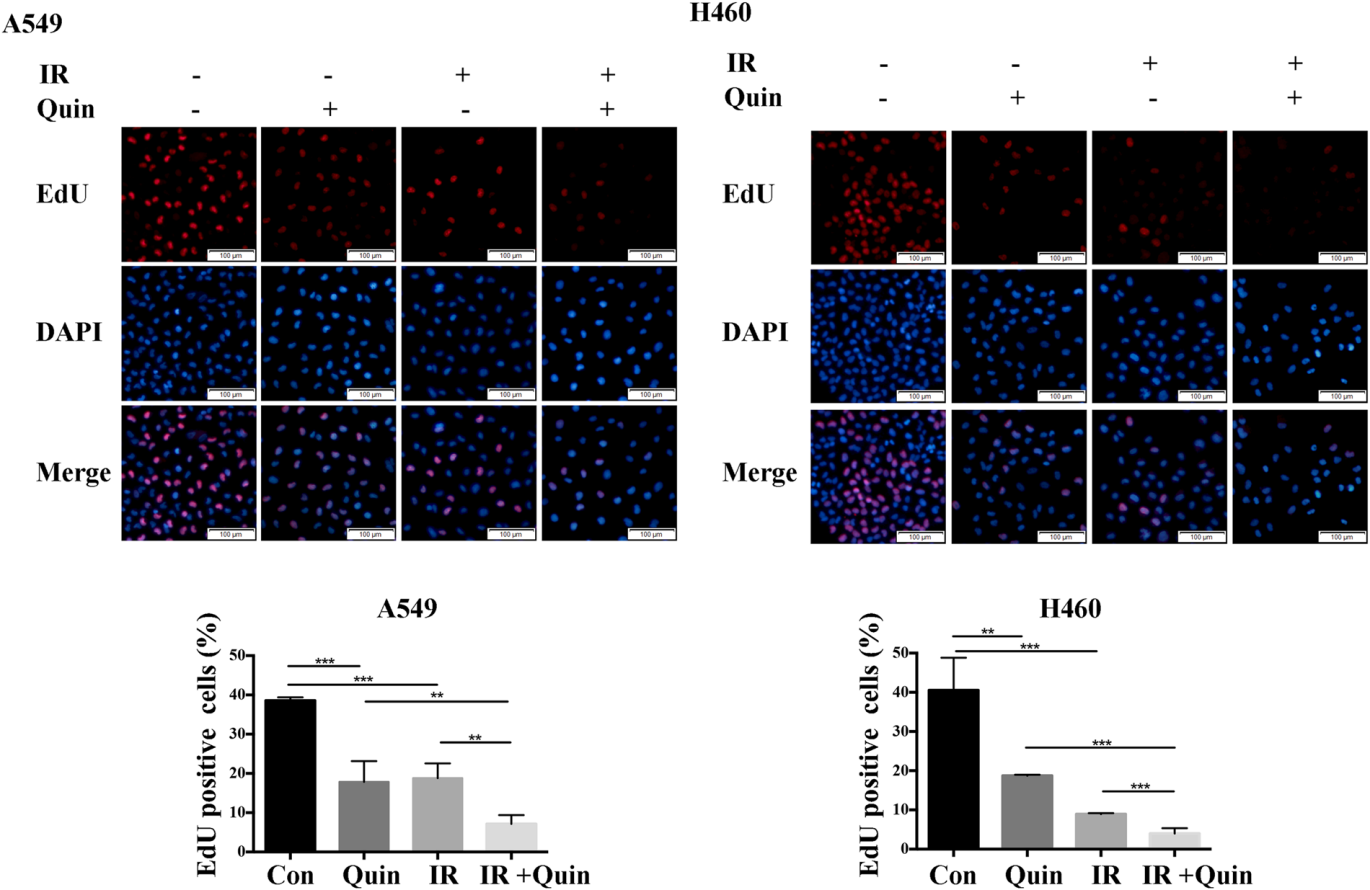
Effect of down regulation of CK2 and IR on cell proliferation. A549 or H460 cells were treated by DMSO or 25 μM Quinalizarin for 24 h and exposed to 0 or 4 Gy X-ray radiation. 24 h after IR, EdU was added for 2 h, and the cells were fixed, permeabilized, labeled with Apollo fluorochrome, and the nuclei were stained with DAPI. Fluorescence images were taken at 100 × magnification. The percentage of EdU positive cells were quantified. (**p < 0.01 and ***p < 0.001) The mean ± S.D. was calculated for three independent experiments.

EdU (5-ethynyl-2′-deoxyuridine) can be incorporated into DNA during active DNA synthesis, which means only proliferating cells can be stained by EdU. To analyze the effects of Quinalizarin and IR on cell proliferation, EdU incorporation assays were used in A549 and H460 cells. Figure 6 shows that both Quinalizarin and IR cause an obviously decrease in EdU staining in both A549 (38.57 ± 0.83% vs 17.86 ± 5.28% and 38.57 ± 0.83% vs 18.80 ± 3.76%, respectively) and H460 cells (40.52 ± 8.28% vs 18.72 ± 0.25% and 40.52 ± 8.28% vs 8.95 ± 0.25%, respectively) and that their combination led to a much greater decrease (7.13 ± 2.26% in A549 and 3.97 ± 1.38% in H460). These results indicated that the compared with each treatment alone, the combination of Quinalizarin and IR suppressed DNA replication. Quantitative analysis revealed that this change is statistically significant (p < 0.01, p < 0.001).

### Down regulation of CK2 increases IR induced double-strand breaks (DSBs) and slows down DSB repair

**Figure 7:**
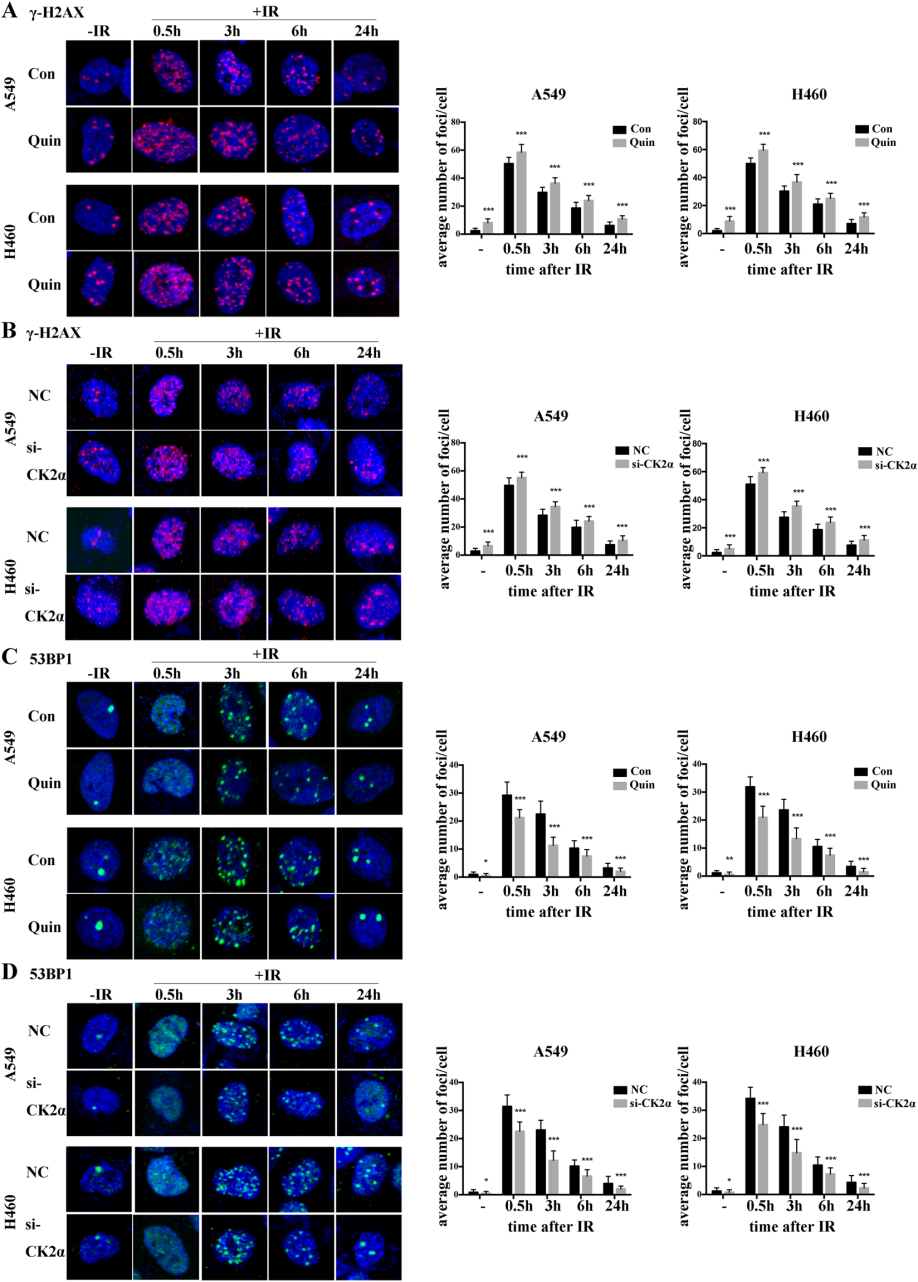
(**A**,**C**) A549 or H460 cells were pretreated with DMSO or 25 μM Quinalizarin for 24 h and then exposed to 4 Gy X-ray radiation. (**B**,**D**) A549 or H460 cells were transfected with negative control siRNA or si-CK2α. After 48 h, the cells were exposed to 4 Gy X-ray radiation. Cells were fixed before IR or 0.5, 3, 6, 24 h after IR, then permeabilized and blocked. Immunofluorescence staining showed the expression of γ-H2AX (red) and 53BP1(green). The cell nuclei were stained with DAPI. Fluorescence images were taken at 400 × magnification by a scanning confocal microscope. The average number of foci per cell were calculated. (*p < 0.05, **p < 0.01, ***p < 0.001) The mean ± S.D. was calculated for three independent experiments.

DNA DSBs are one of the most important therapeutic functions of IR in killing the cancer cells, and the ability to repair DSB influences cell radiosensitivity. To detect the effect of the down regulation of CK2 though Quinalizarin or transfection with si-CK2α on DSB repair, we stained γ-H2AX foci, which appeared immediately when a DSB happens, at different points after 4 Gy X-ray radiation in A549 and H460 cells. As observed in Fig. 7A, the Quinalizarin-treated cells showed more γ-H2AX foci than the DMSO-treated cells without IR, indicating that CK2 depletion can increase DSBs in these two cell lines. At 0.5 h, 3 h, 6 h and 24 h after IR, there were more γ-H2AX foci in the Quinalizarin-treated cells than in the DMSO-treated ones, suggesting that CK2 depletion slowed down DSB repair. The results were confirmed in the si-CK2α-transfected cells (Fig. 7B). At all time points, there were significant differences (P < 0.001).

We also detected 53BP1, which functioned as a molecular scaffold for the recruitment of proteins in DSB repair, in A549 and H460 cells. It was reported that CK2 was necessary for 53BP1 foci formation^18^. Here, we found a significant decrease in 53BP1 foci formation in Quinalizarin-treated cells before IR or at different time points after IR (Fig. 7C). Quantification of 53BP1 foci increased 0.5 h after IR and began to decline after IR over time. However, we observed that after 3 h, 53BP1 foci became larger in size than they were after 0.5 h. Quinalizarin treatment resulted in significant reduction of the foci number at all time points, suggesting that CK2 depletion suppressed DSB repair by affecting the assembly of 53BP1. Similar results were obtained in si-CK2α-transfected cells (Fig. 7D).

### *In vivo* inhibition of down regulation of CK2 on tumor growth

**Figure 8:**
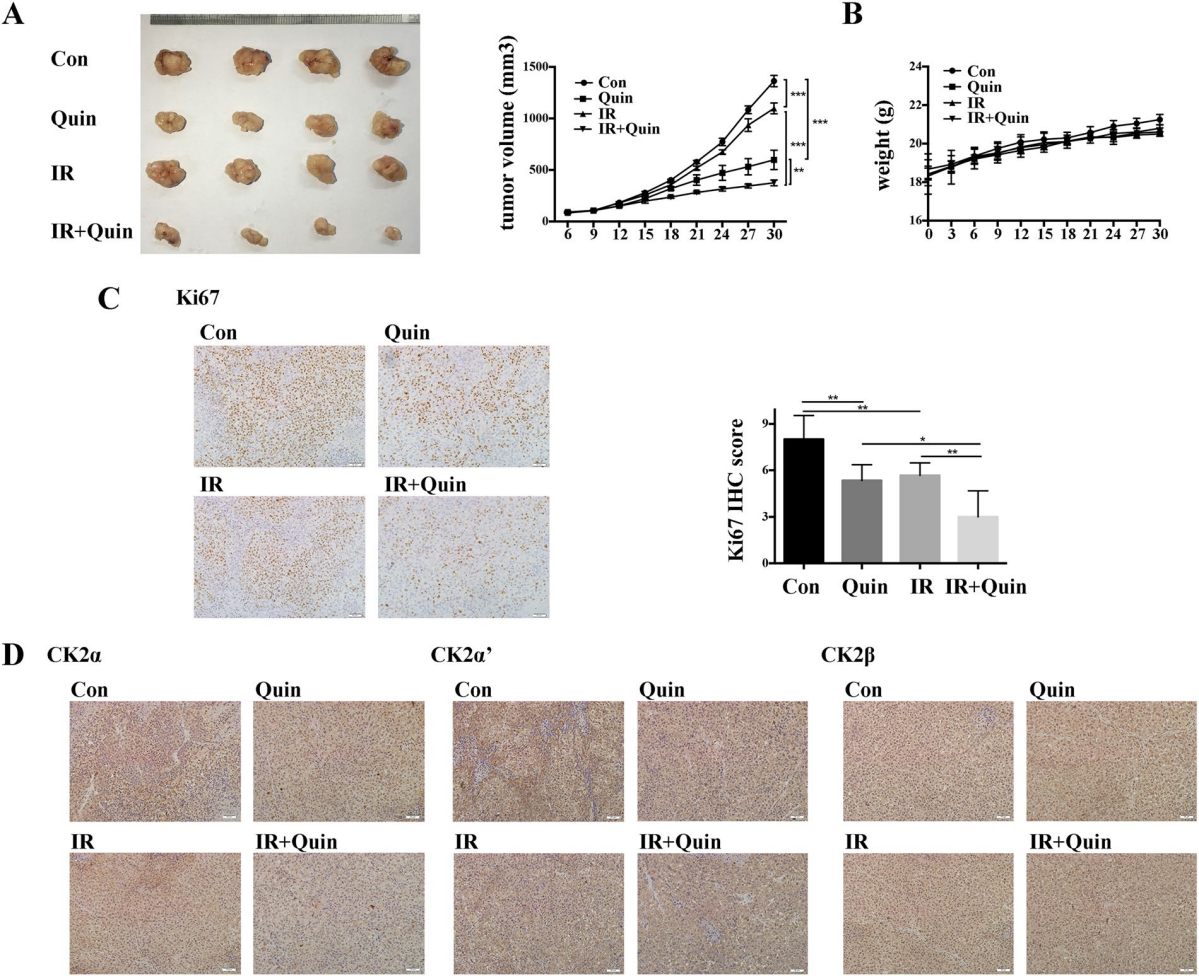
H460 cells (1*10^7^) were inoculated subcutaneously in the right leg of BALB/C nude mice. The volumes of the tumors were calculated every 3 days. DMSO or Quinalizarin was injected intraperitoneally at day 9 (35 mg/kg·d) for 3 consecutive days and twice a week after IR. Tumors were irradiated with 10 Gy X-ray radiation at day 12. (**A**) Photographs of the tumors and growth curves of tumor size. (**p < 0.01, ***p < 0.001) (**B**) The weight of the mice. (**C**) Ki67 was stained using immunohistochemistry. Images were taken at 200 × magnification. The Ki67 IHC were quantified. The dominant staining intensity was scored as: 0 = absent, 1 = minimal, 2 = moderate, 3 = strong. The percentage of positive cells was scored as: 0 =  < 5%, 1 =  ≥ 5–20%, 2 =  ≥ 20–50%, 3 =  ≥ 50%. IHC scores = staining intensity scores × percentage of positive cells scores. (*p < 0.05, **p < 0.01) (**D**) CK2α, α′ and β was stained by immunohistochemistry. Images were taken at 200 × magnification.

*In vivo* studies were conducted on the H460 tumor xenograft mice model. We found that both Quinalizarin and IR suppressed H460 tumor growth, and the inhibitory effect was greater in the combination group (Fig. 8A). As shown in Fig. 8B, Quinalizarin slightly slowed down the increase in mice body weight. Immunohistochemistry staining of Ki67 showed decreased cell proliferation in the combination group compared with that in the solo treatment group, indicating that Quinalizarin had a notable effect on tumor radiosensitivity (Fig. 8C). Figure 8D shows the expression pattern of CK2α, α′ and β after treatment of Quinalizarin or/and IR.

## Discussion

Protein kinase CK2 was one of the earliest discovered kinases in human history, and aroused people’s attention due to its extensive biological roles^8^. As CK2 has been found to be overexpressed in all cancers and is closely related to tumorigenesis, it is considered to be an important target for tumor therapy^5^. The purpose of our study was to investigate the role of the down regulation of CK2 in the radiosensitization of lung cancer cells.

It was reported that CK2α is ubiquitously expressed in different types of lung cancer cells^19^, and the overexpression of the CK2α gene was also reported in lung cancer tissues^20^. In this study, we found higher expression of three individual CK2 subunits in various *in vitro* lung cancer cells than in non-cancerous endothelial cells. In addition, in different pathological lung cancer tissues, the expression of CK2α, α′ or β was also increased compared with that in para-cancerous tissues (Fig. 1). These findings were in consistent with the previous studies and indicated a close association between CK2 and lung cancer.

To explore the impact of the down regulation of CK2 on the radiation response, we employed Quinalizarin, a specific CK2 inhibitor, to inhibit the activity of CK2 *in vivo* and *in vitro* experiments. We also tried si-CK2α to exclude the nonspecific effects of the inhibitor. The data showed that the inhibition of CK2 enhanced the radiosensitivity of nasopharyngeal carcinoma cells^21^, but failed to radiosensitize malignant glioma cells^22^. Our results showed that pretreatment with Quinalizarin before IR decreased SF_2_ in A549 and H460 cells, revealing that Quinalizarin enhanced the radiosensitivity (Fig. 3B). This result is in accordance with Yu-Ching Lin, *et al*. The anthors found that the combination of IR and CK2 inhibitors TBB, TBCA or Hematein can reduce the viability of non-small cell lung cancer cells^19^. In our study, this was the first time that colony formation assays, the gold standard for testing radiosensitivity, were conducted in human lung cancer cells after CK2 inhibition. We also applied si-CK2α and tested small cell lung cancer cells to verify the function of down-regulating CK2 in the radiosensitization of lung cancer cells (Fig. 3D and supplementary Fig. S1). In the *in vivo* experiments, Quinalizarin also showed a radiosensitization function, while compared with IR or Quinalizarin treatment alone, the combination treatment with IR and Quinalizarin apparently reduced the tumor growth (Fig. 8A).

Ionizing radiation can induce DNA double-stand breaks, which cause chromosomal breakage, genomic instability, and lots of complicated cellular reactions^23^. There are two major DSB repair pathways, non-homologous end-joining (NHEJ) and homologous recombination (HR)^24^. The major proteins involved in NHEJ are DNA-PKcs, Ku70, Ku80, DNA ligase IV and XRCC4^25^. It was reported that XRCC4, which acts as a scaffold to recruit DNA ligase IV to the DSB, was a substrate of CK2^12^. MDC1, a critical adaptor protein in HR, can be activated by CK2^13^, promote the recruitment of Rad51 and MRN (Mre11/RAD50/NBS1) and directly interact with γ-H2AX^26^. γ-H2AX appears rapidly and sensitively reflect the DNA double-stand breaks following IR^27^. In the present study, in different human lung cancer cells after exposure to IR, significant increases and delayed removal of γ-H2AX foci were observed in the Quinalizarin or si-CK2α pretreated group compared with those in the control group (Fig. 7A,B). This indicated that down regulation of CK2 increases the IR-induced DSBs and slows down their repair. Our results are in agreement with Xiaofen Pan, *et al*. and Felix Zwicker, *et al*., who found that the inhibition of CK2 increases the number of IR-induced γ-H2AX foci and delays their removal of it in human nasopharyngeal carcinoma cells^21^ and colon carcinoma cells^28^. 53BP1 is a P53 binding protein, which also participants in the early repair of IR-induced DSBs^29,30^. CK2 has been reported to facilitate the recruitment of 53BP1 to DSBs by binding the tandem Tudor domains^18^. Our study observed an apparent decrease of IR-induced 53BP1 foci when treated with Quinalizarin or si-CK2α (Fig. 7C,D).

IR induces cell cycle arrest at the two major DNA damage-dependent cellular checkpoints, G1/S and G2/M. The tumor suppressor protein p53 is one of the key protein mediating the checkpoint pathways^31^. It also acts as an activator of apoptosis and mediates both the transcription-independent and transcription-dependent mechanisms^32,33^. CK2 can phosphorylate p53 at Ser386^14^, and studies have shown that the down regulation of CK2 can inhibit cell proliferation, increase apoptosis and promote IR-induced G2/M arrest^28,34–38^. Our results showed that the down regulation of CK2 significantly increased the IR-induced G2/M arrest (Fig. 5) and inhibited the IR-induced cell proliferation (Fig. 6). However, compared to Quinalizarin alone, the combination of IR and Quinalizarin failed to improve the apoptosis rate (Fig. 4A). Additionally, transfection of si-CK2α increased the IR-induced apoptosis rate (Fig. 4C). This might be due to Quinalizarin’s impact on other kinases. To explain in detail, looking back at the history of development of specific CK2 inhibitors, there are always unknown kinases that could be inhibited by the currently recognized specific CK2 inhibitors. Thus, the radiosensitizing effect of Quinalizarin might not be solely due to the inhibition of CK2, which facilitates our further study using siRNA technology to knockdown the catalytic subunit of CK2 and prove the hypothesis. However, this is also not perfect since the protein-protein interaction between CK2 and other factors, besides kinase inhibition, was missed. Therefore, different results obtained by the two methods could be explained in this regard.

Lung cancer is one of the most common malignant tumors with the highest morbidity and mortality in the world^39^. Because radiotherapy is an important treatment of lung cancer, radiotherapy resistance is a major problem. Our results demonstrated that down regulation of CK2 enhanced the radiosensitivity of lung cancer *in vivo* and *in vitro*, indicating that CK2 inhibitors may be used in the clinic to overcome the radiotherapy resistance in lung cancer.

## Materials and Methods

### Cell culture and reagents

The human lung cancer cells A549, H460, H1975, Hcc827, H446 were acquired from the Chinese Academy of Science Committee on Type Culture Collection Cell Bank (Shanghai, China). All the cells were cultured in 5% CO_2_ at 37 °C in RPMI 1640 (Gibco, Carlsbad, CA, USA) supplemented with 10% fetal bovine serum (FBS, Gibco, CA, USA).

Quinalizarin (Merck, Germany) was dissolved with DMSO to a storage concentration of 10 mM, and diluted with culture media to the indicated concentration immediately before use.

### Western Blot

Cells were harvested, lysed in RIPA containing 1% PMSF (protease and phosphatase inhibitor, Google, Wuhan, China), and the protein concentration was detected by a BCA protein assay (Google). Equal amounts of protein were separated on the SDS-PAGE and then transferred onto PVDF membranes (Millipore, CA, USA). After the membranes were blocked with 5% nonfat dry milk, they were incubated with primary antibodies at 4 °C overnight. The primary antibodies used were as follows: rabbit polyclonal anti-CK2α, α’or β antibodies (the antibodies anti-CK2α #212, anti-CK2α′ #30, anti-CK2β #269 were generated as previously described^40^ and were kind gifts from Prof. Dr. Mathias Montenarh, Universität des Saarlandes, Germany); mouse monoclonal anti-PARP, P53 and α-tubulin (Santa Cruz Biotechnology, CA, USA), anti-β-actin and GAPDH (Abcam, Cambridge, UK). Membranes were washed and incubated with secondary antibody (Invitrogen, NM, USA) at room temperature for 1 h, then visualized using a chemiluminescence kit (Santa Cruz, TX, USA). The intensity of the bands was detected by ImageJ (V1.49t for Mac, National Institutes of Health).

### Immunohistochemistry

Formalin-fixed and paraffin-embedded tumor tissues were cut into sections. After deparaffinization, rehydration, antigen retrieval, blocking of endogenous peroxidase activity and blocking with BSA, the sections were incubated with anti-CK2α, α′ or β (previously described in^40^) and mouse monoclonal anti-Ki67 (Abcam, Cambridge, UK) antibodies overnight at 4 °C. Sections were incubated with secondary antibodies for 1 h and counterstained with hematoxylin for 30 s before dehydration and mounting. Photographs were taken by a light microscope.

### Kinase assay

A549 and H460 cells were treated with DMSO or 25 μM Quinalizarin for 6 or 24 h. The Total protein was extracted. 30 μl of CK2 mix (25 mM Tris-HCl, pH 8.5, 150 mM NaCl, 5 mM MgCl_2_, 50 μM ATP, 1 mM DTT, 0.19 mM substrate peptide), CK2 specific substrate peptide with the sequence RRRDDDSDDD and 10 μCi/500 μl γ[^32^ P] ATP was added into 20 μL of kinase buffer (50 mM Tris/HCl, pH 7.5, 100 mM NaCl, 10 mM MgCl_2_, 1 mM DTT) containing 30 μg of proteins and incubated at 37 °C for 5 min. The mixture was pipetted onto a P81 cation-exchange paper and washed 3 times with 85 mM phosphoric acid and once with ethanol. After the paper dried the Čerenkov-radiation was determined using a scintillation counter.

### CCK8 assay

Cell viability was measured using the cell counting kit-8 (CCK8) assay. Cells were seeded into 96-well plates (2 × 10^4^ cells/well) overnight. After being treated with DMSO or 12.5, 25, or 50 μM Quinalizarin for 24 h, 48 h or 72 h, the cells were incubated with CCK8 solution (Boster, Wuhan, China) for 1 h at 37 °C. Then, the absorbance was detected at 450 nm by a micro plate reader (BioRad, Richmond, CA, USA).

### Colony formation assay

Cells were seeded into 6-well plates, at a density of 200, 400, 600, 800 and 1000 cells per well overnight and treated with different concentrations of Quinalizarin for 24 h or transfected with negative control siRNA or si-CK2α for 48 h, then exposed to 0, 2, 4, 6 and 8 Gy radiation, respectively. A linear accelerator (Primus K, Siemens, Munich, Bayern, Germany) with 6 MV photons/100 cm focus-surface distance with a dose rate of 2.0 Gy/min was used. The culture medium was changed to the fresh media 24 h after IR. After further incubation for 14 days, the cells were fixed with methanol and stained with Giemsa, and the colonies containing not less than 50 cells were counted as clones. The survival curves were fitted according to the multitarget click model, SF = 1 − (1 − e^−D/D0^)^N^ (D, radiation dose; e, the bottom of the natural logarithm; D_0_, the mean death dose; N, extrapolate number; SF, cell survival fraction).

### Down regulation of CK2α by siRNA interference

siCK2α was synthesized as follow: 5′TCC ATT GCT GAA ATG GTA3′. Cells were seeded into 6 well plates overnight. Transfection was conducted using GenMute siRNA transfection reagent according to the manufacturer’s instructions.

### Quantitative RT-PCR

Total RNA was extracted using TRIzal Reagent (Invitrogen) and reverse-transcribed into cDNA using the RevertAid First Strand cDNA Synthesis Kit (Thermo) according to the manufacturer’s instructions. The amplification was performed using the SYBR Prime Script RT-PCR Kit (TaKaRa Bio, Japan) with the Step One Plus Real-Time PCR system (Applied Biosystems).

### Flow cytometric analysis of Apoptosis

Cells were pretreated with DMSO or 25 μM for 24 h, or transfected with negative control siRNA or si-CK2α for 48 h, then exposed to 0 or 4 Gy X-ray radiation. After 24 h, the cells were collected, resuspended in 500 μl binding buffer, then stained with 1 μl Annexin-V-PE and 5 μl 7-AAD (Becton Dickinson, CA, USA), and finally tested on a FACScan flow cytometer.

### Flow cytometric analysis of cell cycle

Cells were pretreated with DMSO or 25 μM Quinalizarin for 24 h, irradiated with 0 or 4 Gy X-ray radiation. Cells were harvested 24 h after radiation, fixed overnight, stained with 1 mg/ml RNase for 30 min at 37 °C and 50 μg/ml propidium iodide (Becton Dickinson, CA, USA) for 30 min on ice and tested on a FACScan flow cytometer. Quantification of the cells in G0/G1, S and G2/M phases was performed by CellQuest software (BD).

### EdU proliferation assay

EdU (5-ethynyl-2′-deoxyuridine), a nucleoside analog of thymidine which can be incorporated into the DNA during replication, was used to test the cell proliferation. Cells were seeded into 96-well plates (4 × 10^4^ cells/well) overnight and treated with DMSO or 25 μM Quinalizarin for 24 h, then exposed to 0 or 4 Gy radiation. 24 h after radiation, EdU (RiboBio, Guangzhou, China) was added for 2 h. Then cells were fixed with 4% formalin, permeabilized with 5‰ Triton-X for 20 min on ice, labeled with Apollo fluorochrome for 30 min, and then the nuclei were stained with DAPI for 10 min. Photographs were taken using a fluorescence microscope. EdU-positive cells were counted using ImageJ (V1.49t for Mac, National Institutes of Health).

### Immunofluorescence staining

Cells were seeded onto cover slips, pretreated with DMSO or 25 μM Quinalizarin for 24 h, or transfected with negative control siRNA or si-CK2α for 48 h, and irradiated with a dose of 4 Gy. The cells were fixed with 4% formalin at different time points after radiation and then permeabilized with 5‰ Triton-X for 20 min on ice, blocked with 5% BSA for 30 min and immunostained with mouse monoclonal anti-γ-H2AX or rabbit polyclonal anti-53BP1(both from Abcam, Cambridge, UK) at 4 °C overnight. After washing with PBS, the cells were incubated with Dylight 594 or 488 (EarthOx Life Science, Millbrae, CA, USA) for 1 h avoid light. Cell nuclei were stained with DAPI for 10 min. Photographs were taken using a scanning confocal microscope (Olympus, Tokyo, Japan). Foci numbers per cell were counted in at least 50 cells under the microscope.

### Animal experiments

4 to 6-week old female BALB/C nude mice were obtained from Beijing HFK Bioscience and housed in a laminar flow hood and in a pathogen free room. H460 cells (1*10^7^) were inoculated subcutaneously in the right leg of the mice. DMSO or Quinalizarin (35 mg/kg·d) dissolved in NS were injected intraperitoneally for 3 consecutive days when the volume of tumors reached 100 mm^3^. Then the tumors were irradiated with 10 Gy X-ray radiation. After IR, DMSO or Quinalizarin was injected twice a week. Tumor volumes were measured every 3 days.

### Ethics statement

All experimental protocols were approved by the Animal Care and Utilization Committee of Huazhong University of Science and Technology (HUST, Wuhan, China) and complied with the guidelines for the welfare and use of animal in cancer research (ad hoc committee of the National Cancer Research Institute, UK)^41^.

### Statistical analysis

Statistical analyses were performed by Prism (6.0c for Mac, GraphPad Software, CA) using T test, and P < 0.05 was regarded as statistically significant. Data are shown as the mean ± SD of at least three independent experiments.

## Electronic supplementary material

supplementary figure
