# Supplementary material for: Association of protein kinase CK2 inhibition with cellular radiosensitivity of non-small cell lung cancer
Source: Sci Rep. 2017 Nov 23;7:16134. doi: 10.1038/s41598-017-16012-1 (PMC5700935; doi:10.1038/s41598-017-16012-1)
Supplement: Supplementary file 1 — supplementary figure [file 41598_2017_16012_MOESM1_ESM.pdf]

Association of protein kinase CK2 inhibition with cellular radiosensitivity of non-small cell lung cancer

Qianwen Li[1], Ke Li[2], Tianyang Yang[1], Sheng Zhang[1], Yu Zhou[1, 3], Zhenyu Li[1], Jinrong Xiong[4], Fangzheng Zhou[1], Xiaoshu Zhou[1], Li Liu[1], Rui Meng[1*], Gang Wu[1*]

1 Cancer Center, Union Hospital, Tongji Medical College, Huazhong University of Science and Technology, Wuhan 430022, China

2 Pharmacy Department, Union Hospital, Tongji Medical College, Huazhong University of Science and Technology, Wuhan 430022, China

3 Department of Nuclear Medicine, Tongji Hospital, Tongji Medical College, Huazhong University of Science and Technology, Wuhan 430030, China

4 Oncology department, The Chinese People's Liberation Army 457 Hospital, Wuhan 430012, China

* co-corresponding authors

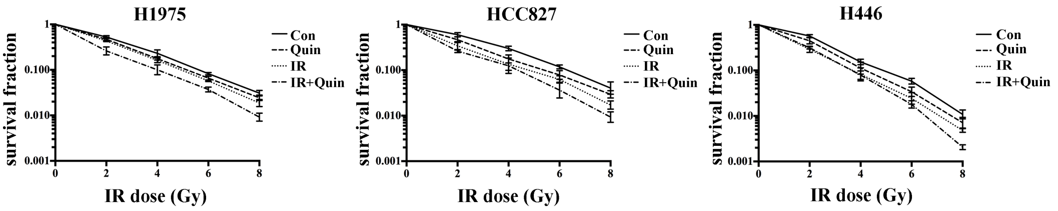

Supplementary Fig.S1 H1975, HCC827 and H446 cells were treated with DMSO or 12.5, 25, 50μM Quinalizarin for 24h, then exposed to 0, 2, 4, 6, 8Gy X-ray. The culture medium was changed to the fresh one 24h after IR. After further incubation for 14 days, radiosensitivity of the cells was measured by colony formation assay. Mean ± S.D. were calculated for three independent experiments.

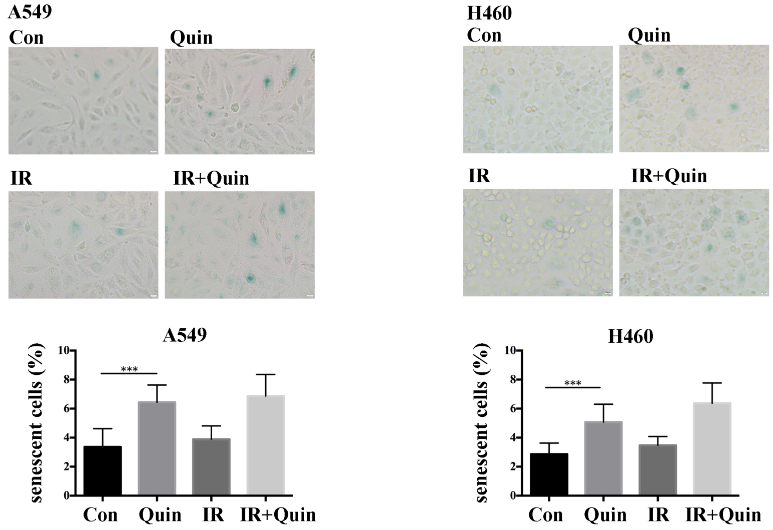

Supplementary Fig.S2 A549 and H460 cells were treated with DMSO or 25μM Quinalizarin for 24h, then exposed to 0 or 4Gy irradiation. After 24h cells were stained for SA-β-gal activity characteristic of senescence. The percentage of senescent cells were quantified. (***p<0.001) Mean ± S.D. were calculated for three independent experiments.
